# Elemental and fatty acid composition of snow algae in Arctic habitats

**DOI:** 10.3389/fmicb.2012.00380

**Published:** 2012-10-29

**Authors:** Elly Spijkerman, Alexander Wacker, Guntram Weithoff, Thomas Leya

**Affiliations:** ^1^Department of Ecology and Ecosystem Modelling, University of PotsdamPotsdam, Germany; ^2^Department of Theoretical Aquatic Ecology, University of PotsdamPotsdam, Germany; ^3^Extremophile Research and Biobank CCCryo, Fraunhofer IBMTPotsdam, Germany

**Keywords:** Arctic snow algal bloom, cellular C:N:P ratio, ecology, extremophiles, lipids, nutrients, psychrophilic, Spitsbergen

## Abstract

Red, orange or green snow is the macroscopic phenomenon comprising different eukaryotic algae. Little is known about the ecology and nutrient regimes in these algal communities. Therefore, eight snow algal communities from five intensively tinted snow fields in western Spitsbergen were analysed for nutrient concentrations and fatty acid (FA) composition. To evaluate the importance of a shift from green to red forms on the FA-variability of the field samples, four snow algal strains were grown under nitrogen replete and moderate light (+N+ML) or N-limited and high light (−N+HL) conditions. All eight field algal communities were dominated by red and orange cysts. Dissolved nutrient concentration of the snow revealed a broad range of NH^+^_4_ (<0.005–1.2 mg N l^−1^) and only low PO^3−^_4_ (<18 μg P l^−1^) levels. The external nutrient concentration did not reflect cellular nutrient ratios as C:N and C:P ratios of the communities were highest at locations containing relatively high concentrations of NH^+^_4_ and PO^3−^_4_. Molar N:P ratios ranged from 11 to 21 and did not suggest clear limitation of a single nutrient. On a per carbon basis, we found a 6-fold difference in total FA content between the eight snow algal communities, ranging from 50 to 300 mg FA g C^−1^. In multivariate analyses total FA content opposed the cellular N:C quota and a large part of the FA variability among field locations originated from the abundant FAs C18:1n-9, C18:2n-6, and C18:3n-3. Both field samples and snow algal strains grown under −N+HL conditions had high concentrations of C18:1n-9. FAs possibly accumulated due to the cessation of growth. Differences in color and nutritional composition between patches of snow algal communities within one snow field were not directly related to nutrient conditions. We propose that the highly patchy distribution of snow algae within and between snow fields may also result from differences in topographical and geological parameters such as slope, melting water rivulets, and rock formation.

## Introduction

Snow algal communities form the macroscopic phenomena of red, orange, and green snow in polar and alpine habitats. Pigmented eukaryotes, inhabiting the snow and imparting the typical color, often consist of potentially mixotrophic green microalgae of the genera *Chlamydomonas*, *Chloromonas*, and *Raphidonema* (Hoham et al., 2002), with the taxon *Chlamydomonas nivalis* widely used as a collective name for species from the former two genera. In addition, filamentous and coccoid cyanobacteria have also been reported in glacier ice cores (Takeuchi et al., 2011) and often dominate the community in cryoconite holes, where they realize high primary production rates (Anesio et al., 2009). Most true snow algal species belong to the Chlamydomonadaceae (Chlorophyta) and are psychrophiles as they tolerate and grow at 0–4°C, and have their optimal growth rates below 15°C (Leya et al., 2009). For a *Chlorella* isolate (Trebouxiophyceae) from a snow field in the Antarctic, optimum growth was reported between 20°C and 25°C (Teoh et al., 2004), thus suggesting this isolate to be cold-tolerant (psychrotrophic or non-obligate cryophilic). Most studies have shown that species diversity is very low in the snow microbial community and that most 18S rDNA originates from fungi (e.g., Bachy et al., 2011).

Snow algal communities have been recognized as an indigenous phenomenon that occurs at certain localities. Field observations and remote sensing analyses have revealed a heterogeneous distribution of algal cells among (Painter et al., 2001) and within snowfields, with the higher densities often observed near the snow line (Takeuchi et al., 2006). Physical factors (slope of snow field, wind, and melting water rivulets) might explain this distribution, as shown in the net ecosystem production of heterotrophic and phototrophic microbes on a Greenland ice sheet that positively correlated with the slope (Stibal et al., 2012). Equally, still little is known about nutrient regimes in algal communities that possibly underlie the occurrence and distribution of red snow (e.g., Newton, 1982; Lütz-Meindl and Lütz, 2006). The involvement of nutrient availability was suggested (Jones et al., 2001), especially because snow algal fields have been found predominantly near bird colonies (Müller et al., 2001). The causes of the heterogeneous distribution within one snow field (local patchiness) have not been fully explained, but also the question as to why snow algae appear on one snow field and not on a neighboring one, remains unresolved. We were therefore interested in nutritional differences in algal snow communities within and between different snow fields.

Snow and ice sheets usually contain very low nutrient concentrations. This applies especially to snow in the Arctic and Antarctic regions because anthropogenic influences are much smaller there (Stibal et al., 2012). The input of nutrients in snow fields is mainly airborne (Newton, 1982), consisting of inorganic and organic dust (= cryoconite; Tazaki et al., 1994), precipitation or sea spray, as well as bird and other animal droppings, whereas near the snow line at the adjoining permafrost soil, additional nutritional input is provided from melt water and vegetation (Tranter and Jones, 2001 and references therein). Small differences in nutrient availability likely results in large effects on algal biomass production and a locally enhanced nutrient patch could thus cause an “algal bloom,” macroscopically visible as the phenomenon of green, orange, or red snow. Green snow normally occurs at the start of the growth season when nutrients are abundant for the algae to mass develop, followed by a color-shift to orange and/or red snow, when algae develop into carotenoid-rich cysts (Hoham and Duval, 2001; Remias, 2012). In many soil and permafrost algae, the development of such resting stages with thickened cell walls in the form of e.g., aplanospores or akinetes, coincides with the accumulation of lipid globules and an increase in secondary carotenoids. This development has been described as a typical reaction to less favorable conditions, such as nutrient depletion (Britton et al., 1998), desiccation and/or high solar radiation (Remias et al., 2005; Leya et al., 2009). Under nitrogen-deficiency, the cell metabolism is directed towards nitrogen-free metabolites, such as fatty acids (FAs) and lipids (Leya et al., 2009). *In situ* nutrient limited conditions have been described for (cyano)bacterial growth in cryoconite holes on glacier surfaces that revealed a (co-)limitation for phosphorus and carbon (Säwström et al., 2007; Stibal et al., 2009) or a nitrogen limitation (Telling et al., 2011).

FAs play an important role in the energy metabolism and overall physiology of algae. They occur in fat or lipid globules, are linked to carbohydrates as glycolipids and are structural and integral parts of the bilayer of membranes, for example, in the form of phospholipids, glycolipids, and betaine lipids (Thompson, 1996). The relative composition of FAs differs between algal species and also strongly depends on environmental factors such as temperature, nutrient availability, and solar radiation (Piorreck et al., 1984; Roessler, 1990). Temperature probably has an important impact on the FA composition of algae and potentially dominates over the impact of other environmental parameters (Poerschmann et al., 2004). Low temperature adapted organisms usually contain a higher ratio of polyunsaturated FAs (PUFAs) to saturated FAs (SFAs). PUFAs are especially incorporated into the lipids of membranes to maintain its fluidity, flexibility, and functionality under cold conditions. In addition, the nutritional regime will change the FA composition of algae (Spijkerman and Wacker, 2011; Piepho et al., 2012), with, in general, a higher total FA content in nutrient limited cultures (Rodolfi et al., 2009).

In general, psychrophilic and psychrotrophic algae will be rich in lipids and in their resting stages lipid globules are often numerous. In the lipid globules, the stored secondary carotenoids, e.g., astaxanthin, are often esterified with FAs. Indeed, red snow algal communities in Antarctica had a higher content of unsaturated FAs (88%) than green snow (72%; Bidigare et al., 1993). The enhanced diversity of the snow algal community with consumers such as ciliates, rotifers, nematodes, ice worms, and springtails (Sattler et al., 2012) most likely enriches the FA composition with long-chained FAs (Desvilettes and Bec, 2009).

To increase our understanding of the ecology of snow algae, we investigated possible nutrient limiting conditions within and among five snow fields along the western coast of Spitsbergen. For a comparison on a species level, the FA composition of four algal strains isolated from snow during previous expeditions was also analysed.

## Materials and methods

**Figure 1:**
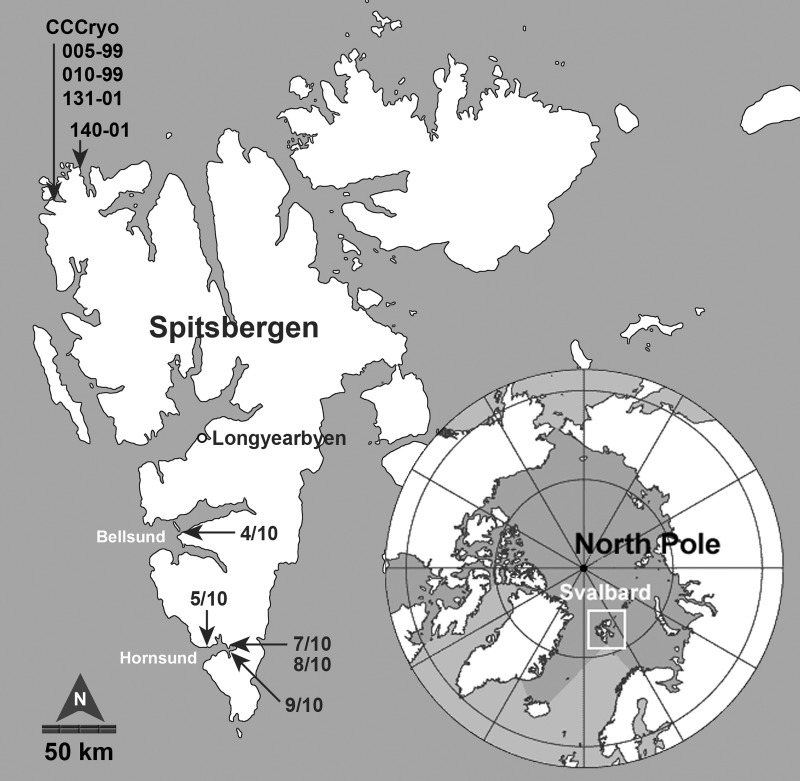
**The archipelago of Svalbard (Norway) is located in the Arctic Ocean, approximately 900 km south of the North Pole.** Field samples were collected at southern locations in the Bellsund and Hornsund area of the main island of Spitsbergen during the KOL 07/2010 expedition (numbered 4/10, 5/10, 7/10, 8/10, and 9/10). The CCCryo strains were originally collected at sites along the northwestern coast in 1999 and 2001. (map insert by Wikimedia Commons, public domain, modified). Scale is approximate.

Analyses were performed on field samples from the KOL 07/2010 expedition and on algal strains obtained from previous expeditions to Spitsbergen (Figure 1). Details regarding geographical origin, locality, and estimated species composition as well as strain origin are listed in Table 1.

**Table 1:** **Description of sampled snow fields and of the site of isolation of algal strains from the Culture Collection of Cryophilic Algae (CCCryo) used for laboratory experiments**.

**Sample**	**Snow color**	**Location name**	**GPS data**	**Locality description**	**Estimated organism composition (field samples) or species name (CCCryo strains)**	**Site orientation**	**Slope (°)**	**Altitude a.s.l. (m)**
**FIELD SAMPLES**								
4/10	Red and orange	Midterhuken	N77.6623° E14.8164°	Steep snow field stretching down to sea level, surrounded by moss vegetation	40% orange cysts, 40% red cysts, 15% transient cells (green, oval, thick walled cysts), <5% cysts of *Cr. brevispina* or *Cr. alpina*, scattered green cells of *Raphidonema* sp. and *Stichococcus* sp.	NNW	45	15
5/10-1a	Red	Hornsund station	N77.0062° E15.5521°	Snow field below bird colony, surrounded by moss vegetation	>95% red cysts, <5% zygotes of *Cr. alpina*, scattered orange cysts and cysts of *Cr. nivalis*; some motile green flagellates	SE	15–20	55
5/10-1b	Orange	Hornsund station	N77.0062° E15.5521°	Snow field below bird colony, surrounded by moss vegetation	>98% orange cysts (7 × 10^5^ cell ml^−1^), scattered red cysts, cysts of *Cr. alpina* and of *Cr. nivalis*, very scattered green cells of *Raphidonema* sp. and *Stichococcus* sp.	SE	15–20	55
7/10	Orange	Adriabukta	N77.0066° E16.2215°	Top of steep snow field surrounded by rocks (no bird cliffs nor vegetation above)	>99% orange cysts, little organic matter, a lot of sediment particles, scattered green cells of *Cr. nivalis*, scattered brown cysts of animal origin?	SW	40	60
8/10	Red and orange	Selbukta	N77.0227° E16.3273°	Snow field on side moraine gravel near sea level (no bird cliffs nor vegetation above)	Red and orange cysts	SW	40–45	5
9/10-1a	Orange	Bautaen	N76.9743° E16.3748°	Intensely orange and red colored snow field on rocky gravel	100% orange cysts	NW	20	75
9/10-1b	Red	Bautaen	N76.9743° E16.3748°	Intensely orange and red colored snow field on rocky gravel	100% red cysts	NW	20	75
9/10-1c	Red and orange	Bautaen	N76.9743° E16.3748°	Wet lower part of above site	40% red cysts without sediment, 20% red cysts with sediment, 30% orange cysts with and without sediment, 5% motile green flagellates, scattered cysts of *Cr. nivalis*; bacteria present, scattered brown cysts of animal origin?	NW	20	75
**CCCryo STRAINS**								
005-99		Bjørnhamna	N79.6500° E11.0000°	Snow field with icy meltwater stream crossing	*Chloromonas nivalis*	NW	5–20	50
010-99		Spitsbergen	n.a.	Snow field	*Chloromonas* cf. *rostafinskii*	n.a.	n.a.	n.a.
131-01		Bjørnhamna	N79.6500° E11.0000°	Snow field with icy meltwater stream crossing	*Raphidonema* cf. *nivale*	NW	5–20	50
140-01		Raudalgeura	N79.7860° E11.8649°	Snow fields near sea level	*Neospongiococcum* sp.	NW	30–40	3

Cr., Chloromonas; n.a., not available; cf., confer (taxonomy not clarified). The average slope of the location was determined by estimating by eye the angle between sea level horizon and snow field top layer.

### Site description and particle characterisation

**Figure 2:**
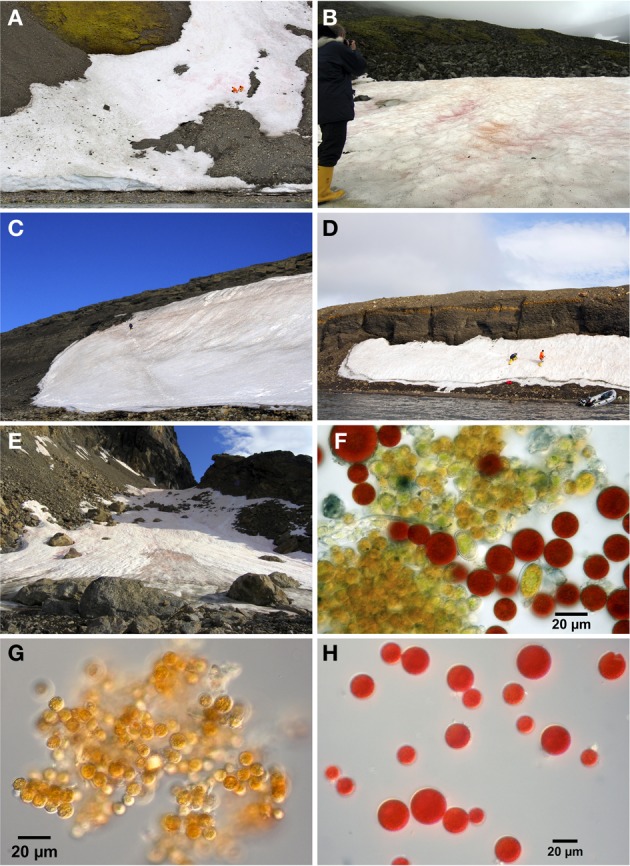
**Different habitat conditions on snow fields at sampling sites. (A)** 4/10 Midterhuken, steep snow field containing mixed orange and red snow algal cysts and with possible nutrient influx from uphill moss vegetation, **(B)** 5/10 Hornsund Station, two differently colored snow algal patches on a flat snow field with possible nutrient influx from moss vegetation and nesting birds. Red tinted snow is visible on the left (5/10-1a) and orange snow in the middle/right of the photo (5/10-1b), **(C)** 7/10 Adriabukta, orange snow consisting of nearly >99% orange cysts surrounded only by rock substrate, **(D)** 8/10 Selbukta, mixed orange and red tinted snow surrounded only by rocky moraine gravel, and **(E)** 9/10 Bautaen, intensely colored patches of orange, red or mixed snow algal communities on a field surrounded by rocks. The orange snow (9/10-1a) is visible in the sun on the left, the red snow (9/10-1b) lies in the shade on the right, and the mixed community (9/10-1c) is the tinted patch in the front of the photo. For detailed information please see Table 1. Microscopic pictures of algal populations: **(F)** diverse algal population consisting of orange and red cysts as well as transient cell stages from different species at site 4/10, **(G)** orange cysts dominating the orange snow patches at site 9/10-1a, and **(H)** red cysts dominating the red snow patches at site 9/10-1b. (For panels **A**,**C**, and **D** we credit Stephan Hering-Hagenbeck).

Eight samples were collected from five snow fields along the western coast of Spitsbergen (Svalbard, Norway) in the Bellsund and Hornsund area between August 4 and 9, 2010 (Table 1, Figure 1). All snow fields macroscopically appeared orange to red in color, a phenomenon which represent snow algal blooms with a mass development of carotenoid-rich algal cysts (Figures 2A–E). Sample sites were selected based on the most contrasting color present, presumably reflecting a different community composition, which for example resulted in the sampling of red and orange snow within one snow field at site 5/10 (Figure 2B). Samples were collected from the uppermost 1–2 cm of intensely tinted areas into a clean bucket or sterile polypropylene containers. After thawing at ambient air temperatures of 0–5°C sub-samples were taken. Sampling sites were characterized by geographical position, site orientation and height above sea level using a GPS device (nüvi 550 all-round, Garmin Deutschland GmbH, Garching, Germany; Table 1).

Fresh field samples were immediately checked under the microscope (Olympus BX40) for species composition and sub-samples were fixed with Lugol solution for later analyses. Field sample 5/10-1b was directly counted using a Thoma Chamber, whereas all Lugol fixed samples were enumerated using an automatic particle counter (CASY 1, Model TT, Schärfe, Reutlingen, Germany). In most samples both small (<7 μm), intermediate (7< μm <12), and larger (>12 μm) sized particles could be distinguished, which coincided with different sized algal cells observed under the microscope (Figures 2F–H). Based on the microscopic observation of 100% orange snow at site 9/10-1a and 100% red snow at site 9/10-1b (Table 1) these two samples were counted and the different size classes defined by estimating the best possible separation in the CASY. Enumeration was performed separately for these size classes. Cell sizes, as measured by the CASY, appeared slightly smaller than under the microscope, partly resulting from dehydration during fixation processes. We therefore did not calculate average cell size.

### Culture experiments

Experiments were performed with four algal strains collected on previous cruises to Spitsbergen, Svalbard (Table 1) that are available at the Culture Collection of Cryophilic Algae (CCCryo, IBMT Potsdam-Golm, Germany). Three stains typically found on arctic snow were selected: *Chloromonas nivalis* (CCCryo 005-99), the tentatively designated *Chloromonas rostafinskii* (CCCryo 010-99), and *Raphidonema nivale* (CCCryo 131-01). The latter strain is a green microalga occurring in green snow. In addition, CCCryo 140-01, a strain most probably belonging to the genus *Neospongiococcum*, and more sporadically found on snow, was used in the experiments. Cultures were grown in Bold's Basal Medium (Starr and Zeikus, 1993) containing triple-concentrated nitrate (9mM N), and this was also used for the standard growth routine at pH 5.5 (3N-BBM). Algae were grown at 4 ± 1°C in a 16:8 light:dark cycle at 150–170 μmol photons m^−2^ s^−1^ (LI-COR light meter LI-250 with spherical sensor US-SQS/L, Heinz Walz GmbH, Effeltrich, Germany) in low temperature plant culture cabinets (Percival Scientific, model I-36(LL)VLX, CLF Plant Climatics, Germany).

After reaching the late log-phase, cultures were gently centrifuged (2200 × g for 5 min. at 4°C) and washed with N-free BBM-medium by centrifugation before the pellet was partly transferred to fresh 3N-BBM medium (+N medium) and partly to nitrate-free BBM-medium (−N medium). The +N-cultures were cultivated under moderate light (ML) conditions as above, whilst the −N cultures were additionally exposed to an increased light intensity of 380–400 μmol photons m^−2^ s^−1^ (HL). The latter (−N+HL) condition was applied to change the green cells into orange and red forms with high contents of secondary carotenoids and lipids presumably reflecting cyst formation. After 14 days, all samples were harvested for analyses of particulate C and N, and FA composition, as described below.

### Macro–nutrient and fatty acid concentrations

Soluble reactive phosphorus (SRP), dissolved ammonium and nitrate concentrations, and particulate phosphorus (pP) were measured in all field samples. Particulate nitrogen (pN), particulate carbon (pC), and FA concentrations were measured in both field and culture samples. In addition, white, uninhabited snow was collected at one sampling site to determine background concentrations of dissolved nutrients. Dissolved nutrient concentrations were determined in filtrate (GF/F filters, Whatman) recovered from samples prepared for particulate nutrient and FA analyses. For pP, melted snow was filtered on polysulfon filters (0.45 μm, Pall Corporation, USA) and digested by K_2_S_2_O_8_ and 0.5 M sulphuric acid in an autoclave for 20 min. Both pP and SRP were measured on a UV/VIS spectrophotometer (UV2401PC Shimadzu, Japan) at 880 nm using molybdate and ascorbate (Murphy and Riley, 1962).

For pN and pC analysis, the melted snow was filtered on GF/F filters (Whatman) pre-combusted at 450°C for 3 h. After drying, pN and pC were determined in an infra-red gas analyzer (EuroVector CHNS-O Elementaranalysator, Wegberg, Germany).

Dissolved nitrate concentrations were measured on the UV/VIS spectrophotometer at 520 nm after addition of sulphanilic acid and 1–Naphthylamine that reduces NO^−^_3_ to nitrite and forms a red dye (Nanocolor® Nitrate Z, Macherey–Nagel, Düren, Germany). Dissolved ammonium concentrations were determined spectrophotometrically at 655 nm after addition of salicylate reagent, hypochlorite and potassium ferrocyanide solution following Bower and Holm-Hansen (1980).

FA samples were collected on a glass fiber filter (GF/F), with approximately 1 mg pC per filter. Lipids were extracted with dichloromethane: methanol (2:1; v:v), transesterified into FA methyl esters (FAME) by resuspension of the dried extract in 3 M methanolic HCl (Sigma-Aldrich Chemie) and incubation for 20 min at 60°C. After repeated extraction with isohexane, the sample was identified and quantified by gas chromatography (6890N Network GC System; Agilent Technologies, Waldbronn, Germany) according to Wacker and Weithoff (2009). A defined concentration of tricosanoic acid methyl ester (23:0ME, Sigma-Aldrich) was used as an internal standard. FAME were detected by a flame ionisation detector (FID) and quantified by comparison with the internal standard and by using multipoint standard calibration curves determined for each FAME, from mixtures of known composition. FAME were identified via known retention times of reference substances (47885-U, Supelco 37 component FAME mix; Sigma-Aldrich). All individual samples were measured at least twice.

### Statistical analysis

Dissolved and particulate nutrient content and FA composition data from the different localities of snow fields and snow algal cultures were statistically analysed by multivariate data analysis in R2.11.1®. Percentages of FAs were arcsin transformed before analysis. We included the long chain FAs in our analysis, although their contribution was relatively small.

## Results

**Figure 3:**
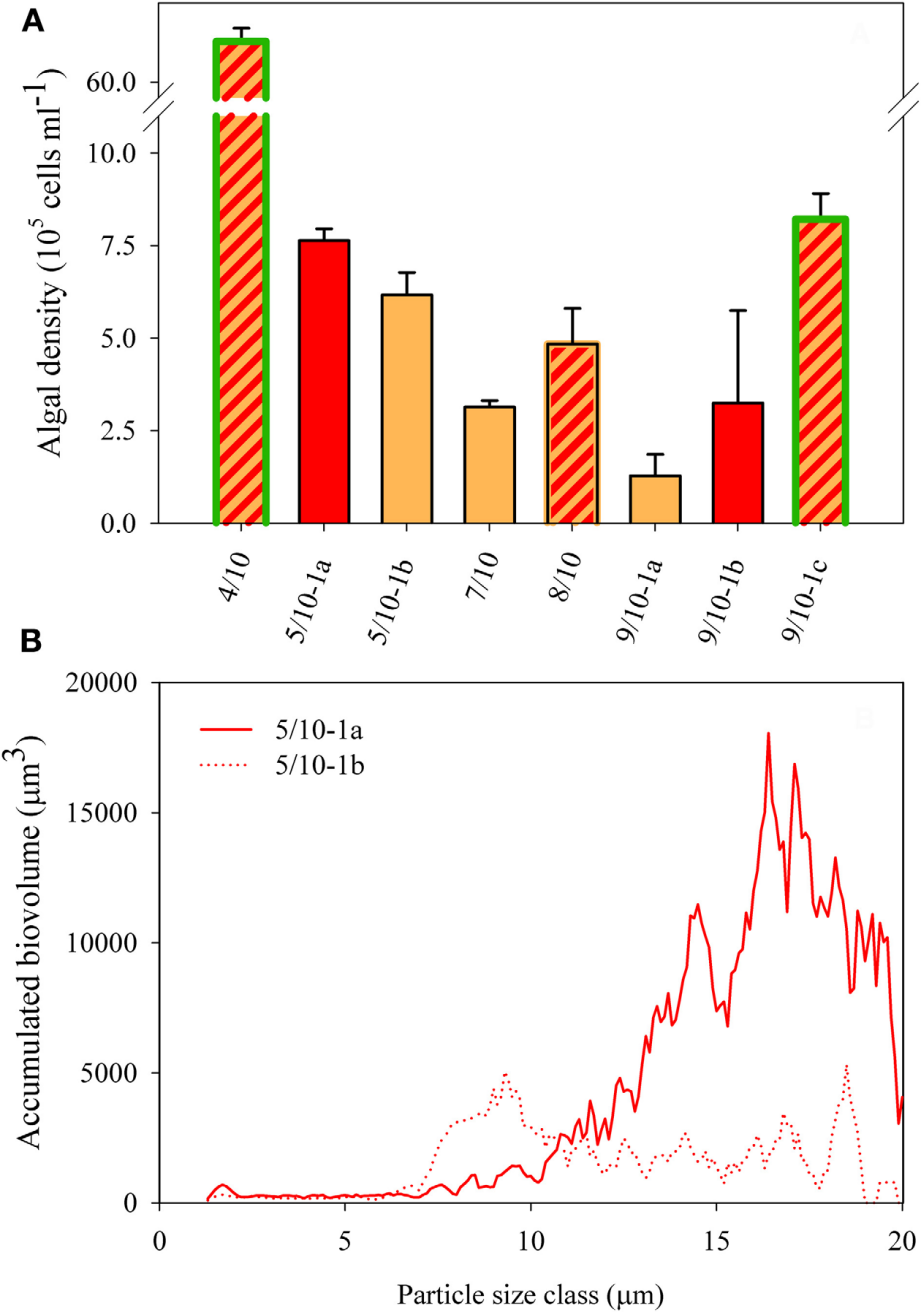
**(A)** Algal cell density as defined by all particles >7 μm in diameter in all field samples. The SD shows the variation of counts within one sample. Color fill of the different bars reflect the color of snow community listed in Table 1. Highlighted green edges of bars indicate the presence of green cells. **(B)** Accumulated biovolume (= density × cell volume within a single particle size class) over particle size at site 5/10-1a and 5/10-1b.

All eight localities consisted of algal communities dominated by red and orange cysts (Figures 2F–H), with only a maximum of ~20% green forms at site 4/10 and ~5% at site 9/10-1c (Table 1). Algal cell densities (as defined by all particles >7 μm in diameter) in the snow algal bloom ranged between 1.3 × 10^5^ and 6.8 × 10^6^ cells ml^−1^ (Figure 3A). The fresh cell counts of the orange snow at site 5/10-1b resulted in a density of 7.0 × 10^5^ cells ml^−1^, which compared nicely with the density in the Lugol fixed sample. There was no clear relationship between the color of snow and algal cell density, although, the highest cell density was measured in the snow with the highest contribution of green forms (site 4/10; Figure 3A).

Microscopically, different sized algal cells could be observed (Figures 2F–H) ranging between 5 and 20 μm in diameter. In the CASY, we observed 2 peaks of algal cells: a peak of intermediate sized particles (between 7 and 12 μm in diameter) and a peak of large sized particles (>12 μm in diameter) which size classes were defined based on samples from site 9/10 (see “Materials and Methods”). Also, in more mixed communities, as present at site 5/10, for example, the size distribution coincided with the microscopic and color observations, with predominantly intermediate sized cells present in orange snow at 5/10-1b and larger sized cells in the red snow of 5/10-1a (Figure 3B). However, estimates of mean cell size did not correlate with the color of snow (not shown).

**Figure 4:**
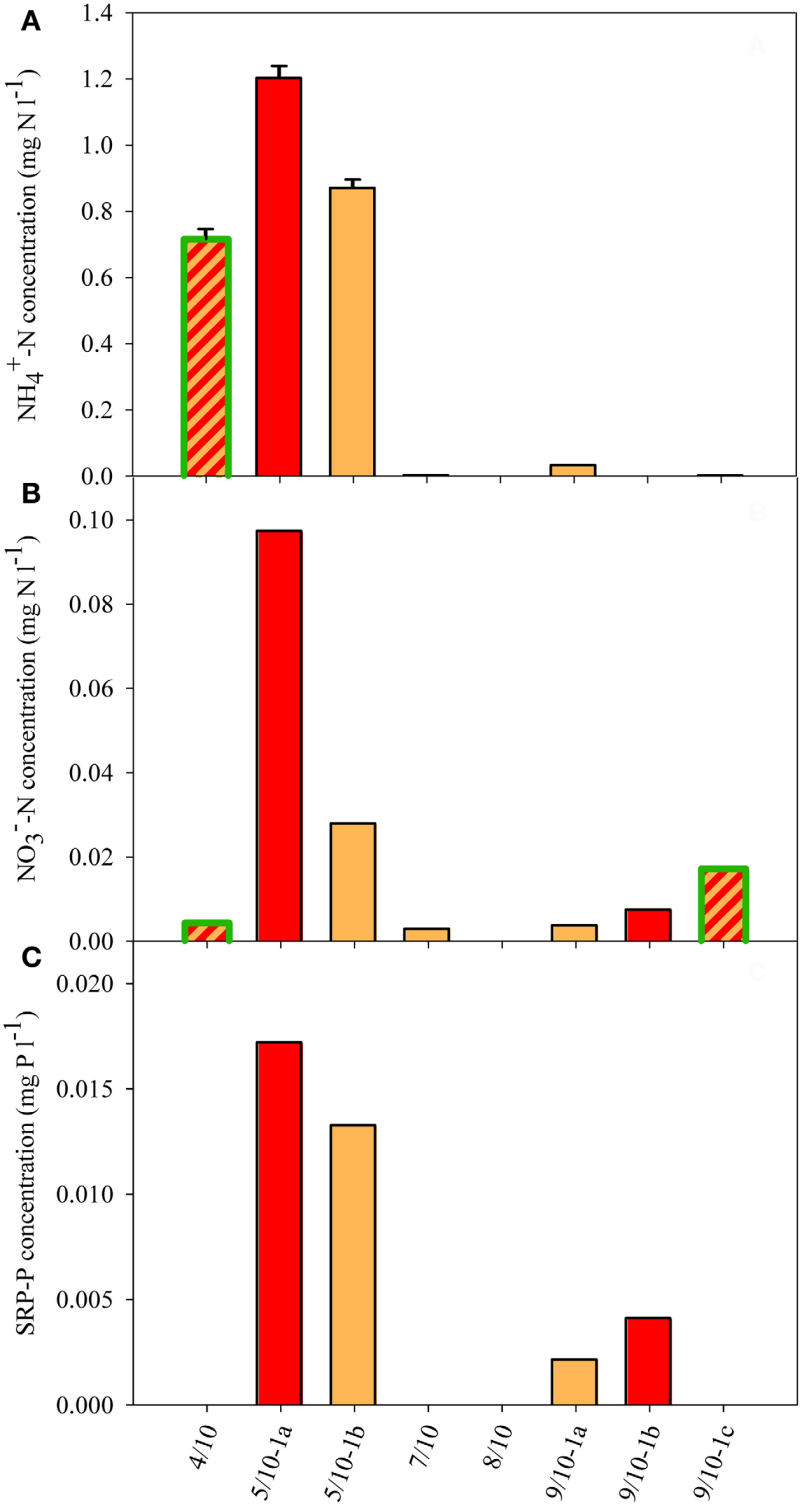
**Dissolved nutrient concentration in the snow of eight sample sites. (A)** NH^+^_4_, **(B)** NO^−^_3_, and **(C)** SRP concentrations. If no value is shown, no concentration could be detected. The SD shown in panel **(A)** reflects the variation resulting from dilution. White snow contained 0.002 mg NH^+^_4_−N l^−1^, 0.002 mg NO^−^_3_−N l^−1^, and 0.002 mg SRP-P l^−1^. Color fill of the different bars reflect the color of snow community listed in Table 1. Highlighted green edges of bars indicate the presence of green cells.

Dissolved nutrient analyses of the snow revealed a broad range of NH^+^_4_ (ranging from <0.005 to 1.2 mg N l^−1^) and low NO^−^_3_ (<0.1 mg N l^−1^), and PO^3−^_4_ (<18 μg P l^−1^) concentrations (Figure 4). There were large differences between the different sites as NH^+^_4_ concentrations were largely enhanced at sites 4/10 and 5/10 and were otherwise only above the detection limit at site 9/10-1a (Figure 4A). The high NH^+^_4_ concentrations at sites 4/10 and 5/10 coincided with the presence of moss vegetation in the direct surrounding of the snow field (Table 1). NO^−^_3_ concentrations were only enhanced at site 5/10-1a, whereas concentrations at 5/10-1b were as low as the lowest concentration of the concentration range (i.e., 0.03 mg N l^−1^; Figure 4B). Nitrate was also detected at site 9/10-1b and 9/10-1c, although only in traces (0.007 and 0.017 mg N l^−1^, respectively). SRP concentrations were all low, and only concentrations at site 5/10-1a and 5/10-1b were at the first concentration of the concentration range (i.e., 0.016 mg P l^−1^; Figure 4C). Concentrations of SRP at site 9/10-1a and 9/10-1b were just traces and did not differ from the concentration determined in white snow (0.002 mg P l^−1^). In general, measurable concentrations of all macro-nutrients could only be determined at site 5/10-1a.

**Figure 5:**
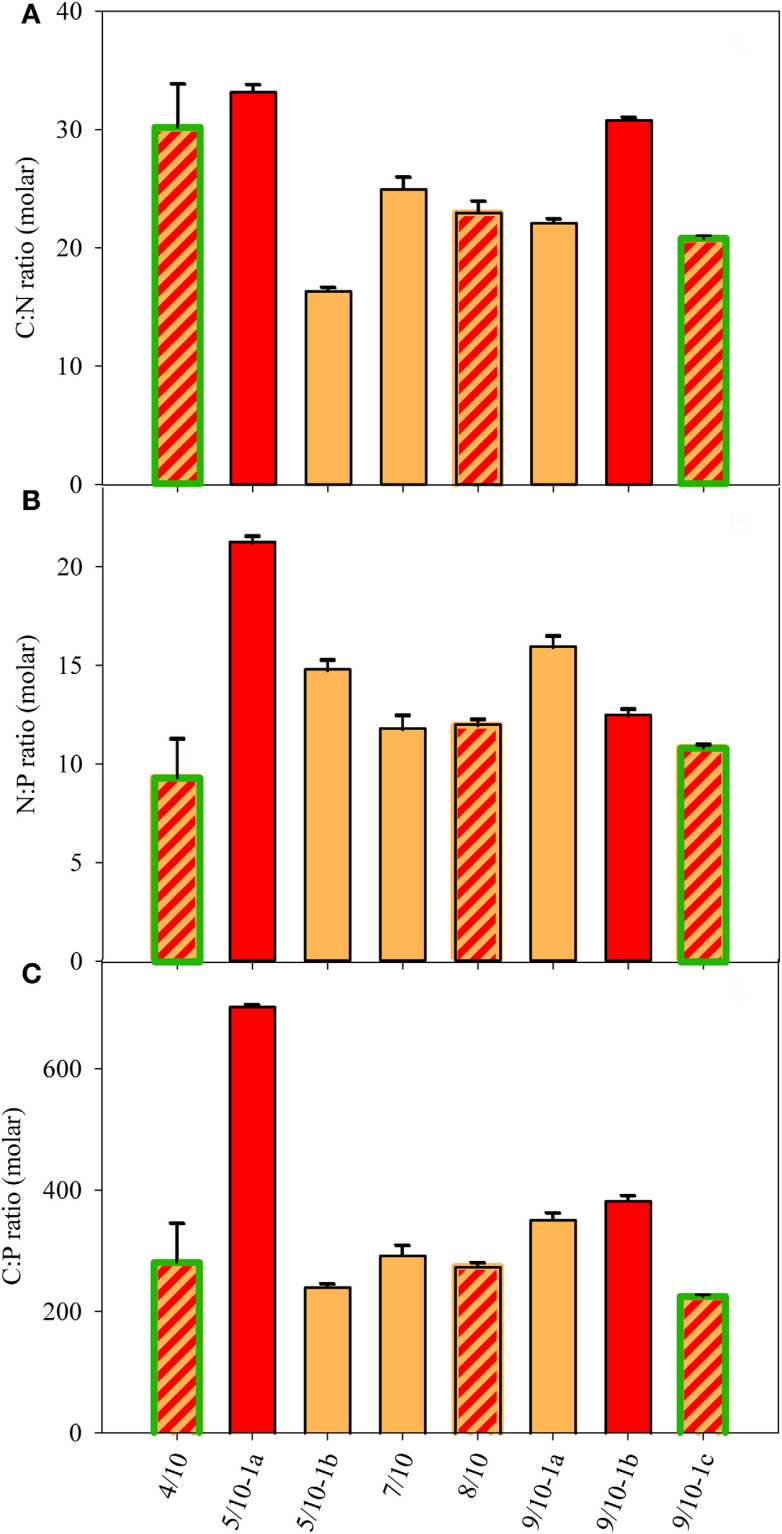
**Particulate nutrient ratios in the snow of eight sample sites (in molar). (A)** C:N ratio, **(B)** N:P ratio, and **(C)** C:P ratio. The SD was calculated from repeated measurements of sub-samples for pC, pN, and pP. Color fill of the different bars reflect the color of snow community listed in Table 1. Highlighted green edges of bars indicate the presence of green cells.

The dissolved nutrient concentrations (Figure 4) did not reflect the particulate nutrient quota of the algae, with molar C:N ratios ranging between 16 and 33 and N:P ratios ranging between 11 and 21 (Figures 5A,B). The N:P and C:P were highest at the site 5/10-1a, which also contained the highest dissolved nutrient concentrations. The higher external concentration of NH^+^_4_ at sites 4/10 and 5/10 did not result in a consistent pattern of lower C:N or higher N:P ratios at those sites. The molar C:P ratio ranged between 220 and 380 at most sites and was highest (700) at site 5/10-1a (Figure 5C). This high value possibly reflected a high cellular carbon content, rather than a low cellular P content as SRP concentrations were highest at this site (Figure 4C). In general, no relation between the external and internal macro-nutrient concentrations or cell volume and snow color was detected at any of the sampled locations.

**Figure 6:**
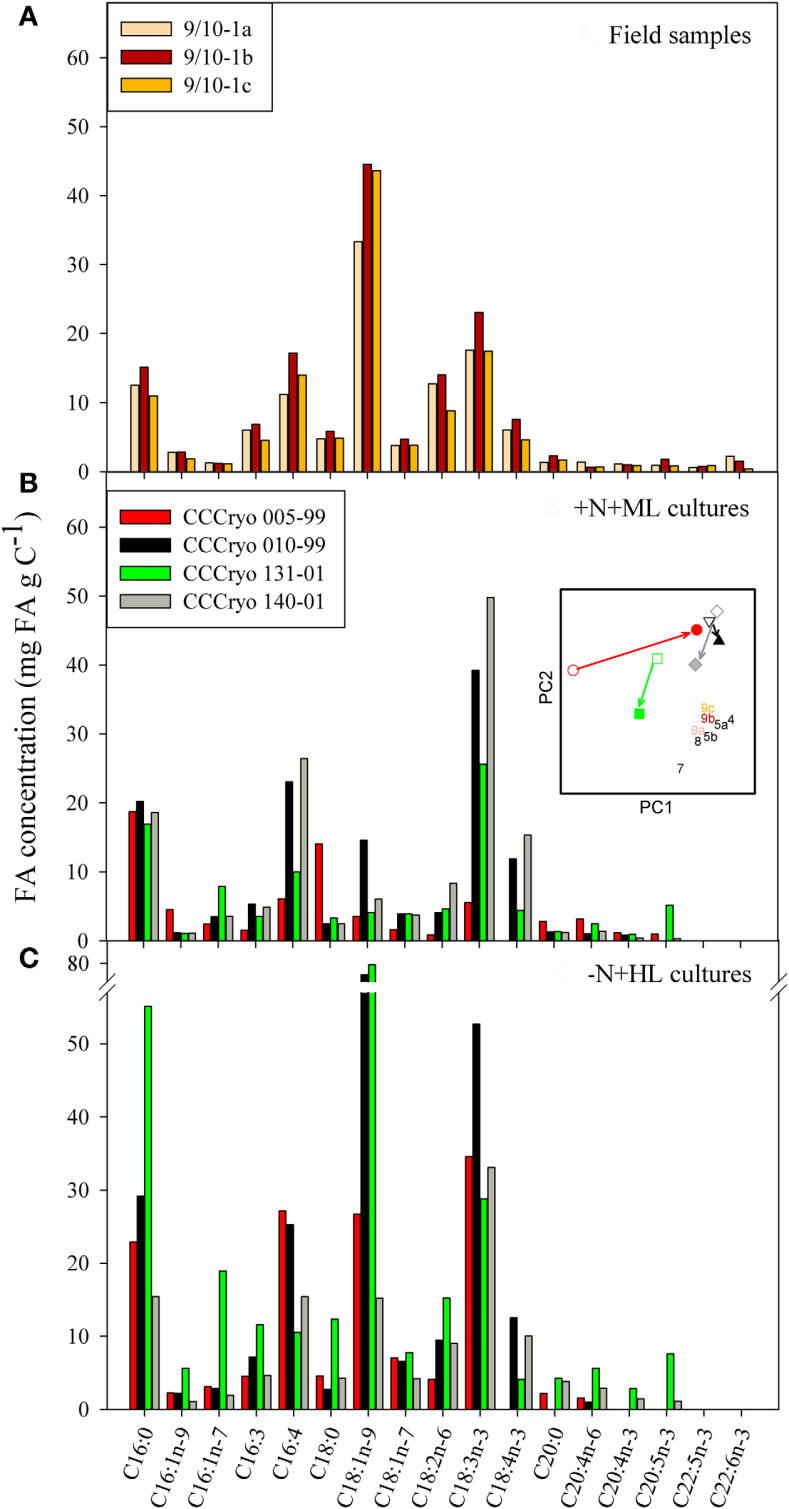
**Compilation of the most abundant fatty acids in (A) field samples 9/10-1a, 9/10-1b, and 9/10-1c and of four snow algal strains cultured under (B) N-replete and moderate light (+N+ML) and (C) N-limiting and high light (−N+HL) conditions.** The inserted figure shows a PCA combining the FA composition of field samples (numbers similar to those in Figure 7 and color of numbers as used in panel **A**) and +N+ML cultures (open symbols) and −N+HL cultures (closed symbols). The colors of the symbols used for the strains are those used in panels **B** and **C**, and different symbols were used to distinguish between the different strains (circles for 005-99; triangles for 010-99; squares for 131-01; and diamonds for 140-01. Arrows illustrate the direction of change from +N+ML to −N+HL conditions.

The general composition of FAs was largely similar between orange and red snow, as exemplified for the orange snow at site 9/10-1a, the red snow at site 9/10-1b, and snow containing a mixed community (9/10-1c; Figure 6A). The mono-unsaturated FA (MUFA) C18:1n-9 was the most abundant FA (Figure 6A). Concentrations of this MUFA reached 45 mg FA gC^−1^ at site 9/10-1b. Other abundant FAs were C16:0, C16:4, C18:2n-6, and C18:3n-3 (α-linolenic acid). The FA composition of the +N+ML laboratory cultures largely differed from the pattern observed in the field samples (Figure 6B), mainly in the 5-fold lower concentrations of the MUFA C18:1n-9 in the laboratory strains. Another striking difference was the absence of the long-chained PUFA C22:5n-3 (docosapentaenoic acid) and C22:6n-3 (docosahexaenoic acid) in all, +N+ML as well as −N+HL, four algal strains (Figures 6B,C). These PUFAs were important constituents in some field samples as they accounted for approximately 0.3–1.7% of the total FA concentration at location 9/10. The most abundant FA in the +N+ML cultures was C18:3n-3 with concentrations up to 50 mg FA gC^−1^ in CCCryo 140-01 (Figure 6B). Other abundant FAs were C16:0, C16:4, and C18:4n-3 (stearidonic acid), although the latter FA was not detected in the *Chloromonas* strain CCCryo 005-99, in contrast to its presence in the other *Chloromonas* strain (CCCryo 010-99). Interestingly, strain CCCryo 131-01 contained rather high concentrations of C20:5n-3 (eicosapentaenoic acid), whereas this PUFA was barely detectable in the other three strains, which is possibly due to its different taxonomic position within the Trebouxiophyceae.

The absolute FA concentrations and the FA composition changed considerably when algae were N-limited and grown at HL (Figure 6C). With the exception of CCCryo 140-01, the total FA (FAtot) concentration increased in −N+HL compared with +N+ML conditions (Table 2). Although the FA concentrations of the different strains changed in a species specific manner, the concentration of the MUFA C18:1n-9 increased in all strains. In CCCryo 010-99 and CCCryo 131-01 this resulted in higher concentrations of C18:1n-9 than the concentration of the PUFA C18:3n-3, thus resembling the general FA composition pattern of the field samples (Figure 6A). To illustrate this resemblance in FA composition, a principal component analysis (PCA) was inserted into Figure 6, showing the distribution of data points based on the relative contributions of the FAs to the total FA content. In this insert, field samples were all grouped together (i.e., they had a largely similar FA composition) and laboratory cultures were scattered to the left and upper part of the graph. The arrows between +N+ML and −N+HL conditions reveal that the FA composition in all strains were heading toward that of the field samples: CCCryo 005-99, mainly over the first axis, and the other three strains over the second axis.

**Table 2:** **Total fatty acid concentrations (FAtot, in mg FA g C^−1^) and contribution of different fatty acid groups to FAtot (SFA, MUFA, and PUFA in %) in the snow samples and four snow algal strains cultured under N-limiting and high light (−N+HL) and N-replete and moderate light (+N+ML) conditions**.

		**FAtot**		**SFA/tot**		**MUFA/tot**		**PUFA/tot**	
**Field**	4/10	294		13.2		33.1		53.7	
	5/10-1a	159		15.2		28.8		56.0	
	5/10-1b	97		16.6		36.7		46.7	
	7/10	55		26.4		24.9		48.7	
	8/10	85		19.6		37.1		43.4	
	9/10-1a	132		18.0		33.5		48.6	
	9/10-1b	161		17.8		34.2		48.0	
	9/10-1c	128		16.9		40.5		42.6	
		**−N+HL**	**+N+ML**	**−N+HL**	**+N+ML**	**−N+HL**	**+N+ML**	**−N+HL**	**+N+ML**
**Culture**	CCCryo 005-99	159	81	25.0	54.4	27.8	18.6	47.1	27.0
	CCCryo 010-99	235	142	17.3	20.8	35.5	18.5	47.2	60.7
	CCCryo 131-01	305	108	29.4	25.7	41.4	18.7	29.2	55.6
	CCCryo 140-01	142	155	19.6	16.8	18.7	11.4	61.7	71.8

Total FA concentrations covered a similar range in both, field and culture samples, and varied between 55 and 300 mg FA gC^−1^ in field samples, and between 80 and 305 mg FA gC^−1^ in cultures (Table 2). The main FAs in the field samples were PUFAs, contributing between 45 and 55% of total FA concentrations. MUFAs were the second largest FA pool, contributing between 25 and 40% of total FA concentrations. Unsaturated FAs were therefore the predominant FAs in the snow algae, accounting for 75–85% of total FA concentrations.

Changes in PUFA and MUFA content between +N+ML and −N+HL conditions differed between strains (Table 2). CCCryo 010-99, CCCryo 131-01, and CCCryo 140-01 predominantly consisted of PUFAs under +N+ML conditions (55–70%), whereas CCCryo 005-99 mainly consisted of SFAs (55%). When grown at −N+HL, CCCryo 010-99 and CCCryo 140-01 maintained their high PUFA content, whereas CCCryo 131-01 nearly doubled its MUFA content to 40%. The changed FA composition at −N+HL did not relate to the increase in the total FA concentration in all strains, except CCCryo 140-01. In general, unsaturated FAs were the predominant FAs in the −N+HL algae accounting for 70–85% of total FA concentrations.

**Figure 7:**
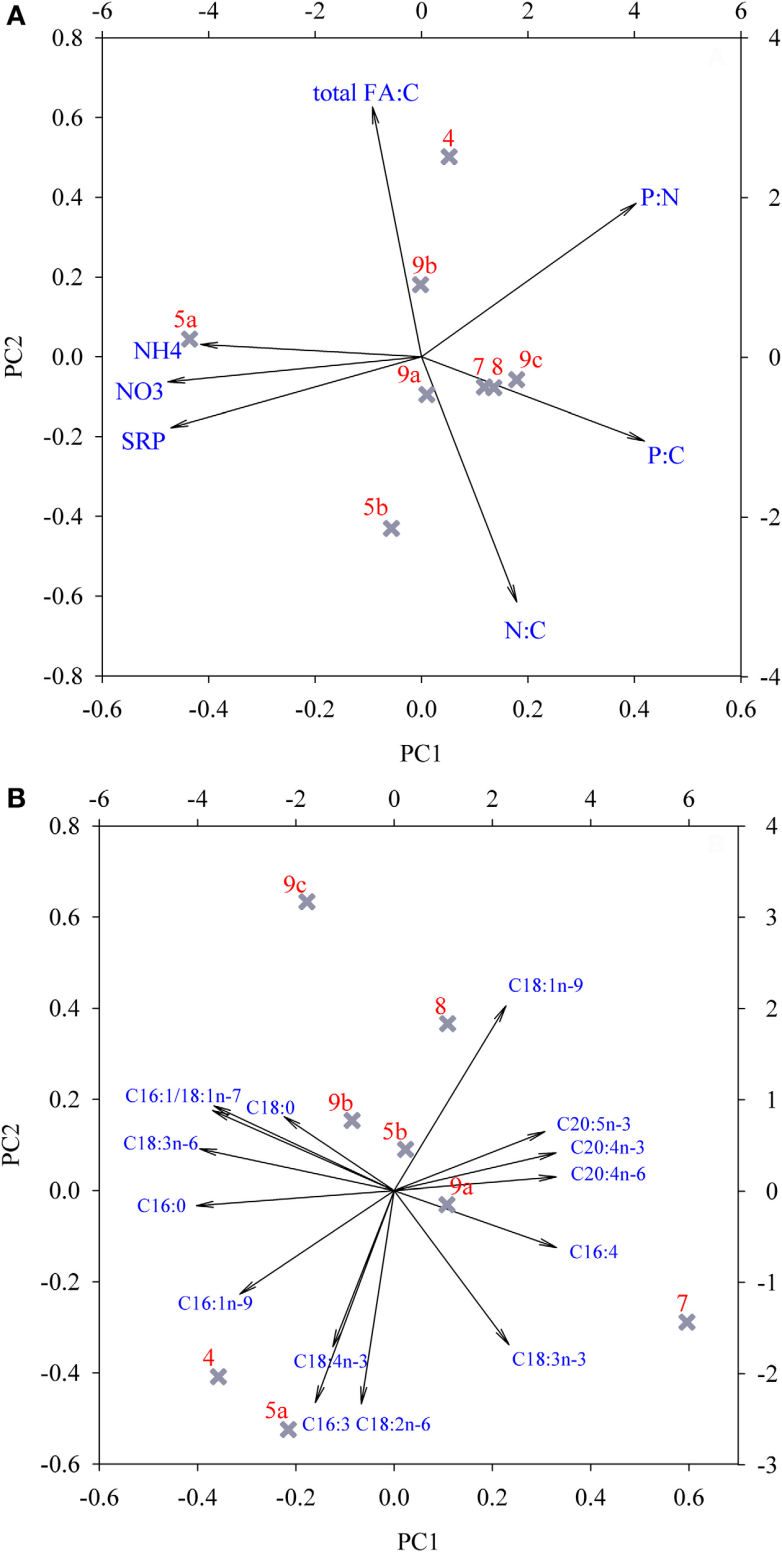
**Principal component analysis of (A) the concentration of macro-nutrients in snow, cellular nutrient quota (i.e., the inverse of C:N:P ratio), and total FA concentrations in snow organisms and (B) separate fatty acid species proportions at all studied locations.** For clarification, site descriptions were abbreviated as follows: 4/10 = 4; 5/10-1a = 5a; etcetera. See Table 3 for underlying values.

The relationships between the concentrations of dissolved macro-nutrients, nutrient ratios of snow organisms and total FA concentration at the different locations were examined with a PCA (Figure 7A). The PCA extracted three factors with eigenvalues >1, which explained 94.1% of total variance (Table 3). PC1 separated location 5/10-1a from all others as a result of the high dissolved nutrient concentrations, and the high organismal P:C quota (i.e., the inverse of C:P ratio; Figure 7A; Table 3, compared with Figures 4, 5). PC2 was mainly loaded by (opposing vectors of) total FA concentration and the N:C quota, suggesting that concentrations of total FA and C:N ratio positively correlated between the snow algal communities. Location 4/10 with its high total FA content (Table 2) was positively, and location 5/10-1b, negatively, correlated with PC2 (Table 3). Both locations were slightly separated from the other locations by their negative correlations with PC3 that explained nearly 16% of data variance and had high loadings of P:C quota and dissolved NH^+^_4_ concentrations (Table 3, but not shown in Figure 7).

**Table 3:** **Summary of principal component analysis (PCA) of (A) the concentration of macro-nutrients and total FAs in organisms and snow and (B) the specific fatty acid composition at all studied locations (arc-sin-transformed proportions)**.

**A**				**B**			
**Analysis of bulk macro-/micronutrients**				**Analysis of specific FA-composition**			
	**PC1**	**PC2**	**PC3**		**PC1**	**PC2**	**PC3**
Eigenvalue	3.72	1.76	1.10	Eigenvalue	8.46	3.89	1.64
Variance explained (%)	53.1	25.2	15.8	Variance explained (%)	56.4	26	10.9
**COMPONENT LOADINGS**							
Particulate concentration				C16:0	0.32	−0.13	−0.03
Total FA:C	−0.09	0.63	−0.43	C16:1n-9	0.22	−0.3	−0.31
N:C (molar)	0.18	−0.61	−0.41	C16:1n-7	0.33	0.09	−0.07
P:C (molar)	0.42	−0.21	−0.48	C16:3	0.07	−0.49	−0.11
P:N (molar)	0.4	0.38	−0.28	C16:4	−0.27	−0.03	0.41
Nutrient concentration				C18:0	0.22	0.12	0.52
NH_4_ (mg l^−1^)	−0.41	0.03	−0.54	C18:1n-7	0.33	0.08	−0.05
NO_3_ (mg l^−1^)	−0.48	−0.06	−0.05	C18:1n-9	−0.14	0.44	−0.19
SRP (mg l^−1^)	−0.47	-0.18	−0.21	C18:2n-6	−0.02	−0.48	−0.21
				C18:3n-6	0.34	0	0
				C18:3n-3	−0.21	−0.26	0.27
				C18:4n-3	0.08	−0.34	0.5
				C20:4n-6	0.33	0.03	0.1
				C20:4n-3	0.33	0.08	0.08
				C20:5n-3	0.31	0.13	0.16

Only factors with eigenvalues >1 are shown, thus showing the data for the PC3 axis, which is not depicted in Figure 7.

The relationships between the FA compositions at the different locations were examined in more detail with another PCA (Figure 7B). The PCA extracted three factors with eigenvalues >1 that explained 93.3% of the total variance in FA composition between locations (Table 3). A high explanation of the variance was obtained by the first two principal components, with 56% explained by the first and 26% explained by the second PC (Table 3). Site 7/10 was separated from the other sites by a strong correlation to PC1, which was mainly represented by high component loadings for the FAs C16:0, C16:1n-7, C18:1n-7, and C18:3n-6 and the long-chain PUFAs C20:4n-6, C20:4n-3, and C20:5n-3 (Table 3). In most samples, the contribution of these three long-chain PUFAs was ≤1%, although at site 7/10, contributions of all 3 PUFAs were between 1.0 and 2.5% of total FA content. Sites 4/10 and 5/10-1a are opposite to sites 8/10 and 9/10-1c regarding PC2, which was mainly represented by C16:3, C16:1n-9, C18:1n-9, and C18:2n-6 but also by C18:3n-3 and C18:4n-3. The FA composition at the locations 4/10, 5/10-1b, and 8/10 were correlated with C18:0 and C18:4n-3 (PC3, Table 3). However, PC3 contributed only 11% to the explanation of the data variance (data not presented).

## Discussion

Eight arctic snow sites visually inhabited by microorganisms (red and orange snow) were sampled to study the macro-nutrient and FA composition. In addition, we measured the FA composition of four snow algal strains cultured under +N+ML and −N+HL conditions. Data revealed a high diversity within one snow field for the color of snow (and thus a patchy distribution of species diversity), which was not related to differences in dissolved and cellular nutrient concentrations or FA composition. The FA composition of the snow algal communities roughly compared with those of −N+HL strains, despite the occasional presence of high dissolved NH^+^_4_ concentrations.

### Population densities and size distribution

In field samples of snow algal blooms we found algal densities ranging between 1.3 × 10^5^ and 6.8 × 10^6^ cells ml^−1^. These values are well within the range of massive blooms of 1 × 10^6^ cells ml^−1^ of *Chlamydomonas nivalis* observed in snow water of cold alpine regions (Sattler et al., 2012). Additionally, cell counts of samples from intensely orange or red tinted snow fields from earlier expeditions to Spitsbergen (KOL 07/1998) ranged between 4 × 10^4^ and 2.1 × 10^6^ cells ml^−1^ (Müller et al., 1998). Values in a similar range were reported in the SW of Svalbard, from the glacier Werenskiold, where cell densities of orange spores in the snow field during the summer of 2004 ranged between 1 and 10 × 10^5^ cells ml^−1^ (Stibal et al., 2007). Cell densities that were exceptionally high were only found at the location, 4/10, which might have resulted from a more efficient algal bloom sampling at this location. Due to the high patchiness of snow algal cysts on the snow field surface, quantitative sampling was not easy. As we usually took samples from the most intensely colored patches, the cell densities stated should be considered as a maximum concentration, rather than as an average of the total patch of snow covered by algal cysts. Although population densities did not correlate with the dissolved nutrient concentrations, the high proportion of >15% green forms (Table 1) at site 4/10 suggested that this was a productive site.

In most field samples we could distinguish three different size classes of particles. The medium-sized peak ranged between 7 and 12 μm in diameter, which mainly originated from orange cysts, and also a fraction of smaller red cysts, of the collective taxon *Chlamydomonas nivalis* (see Figures 2F,G). In comparison, orange spores from the Werenskiold snow field had a mean diameter of 10.2 μm (Stibal et al., 2007). The larger-sized particles were predominantly red cell stages of *Chlamydomonas nivalis* that can measure 20–30 μm in diameter (Stibal et al., 2007; Sattler et al., 2012). The size distribution of the algal cells largely coincided with the predominant color of the snow and microscopic observations. Resting stages from *Chlamydomonas nivalis* have the typical dark red color due to massive astaxanthin accumulation (Remias et al., 2005). Although the red cysts were typically part of the largest particle fraction in our spectrum, smaller red cysts were also present (Figure 2H). At site 4/10 the presence of *Raphidonoma* sp. was observed microscopically: a genus not capable of synthesizing secondary carotenoids and not known to produce cysts (Leya et al., 2009). This filamentous species might have been part of both the medium-sized and the larger-sized peak, depending on the size of the filaments.

### Dissolved nutrients

Large differences in NH^+^_4_ concentrations and, in general, rather low concentrations of NO^−^_3_ and PO^3−^_4_ were measured in different snow fields inhabited by different snow algal communities. Against expectation, no clear differences in dissolved nutrient concentrations between the different colored snow communities were observed. Compared with NH^+^_4_ concentrations in the snow of Cayuse Pass, WA, USA, concentrations at site 4/10 and 5/10 were 20-fold higher, whereas NO^−^_3_ and SRP concentrations were 10-fold lower (Hoham and Mullet, 1977). Concentrations of nutrients that were measured during the KOL 07/1998 expedition to snow fields located in the north of Spitsbergen (Müller et al., 1998) were comparable to those reported here, and SRP values at the site containing the highest concentrations (5/10) were similar to those reported for the Werenskiold snow field in the SW of Spitsbergen (i.e., 10 μg P l^−1^, Stibal et al., 2009). Therefore, the variation in dissolved nutrient concentrations from year to year appears to be small along the western-coast of Svalbard, and locally enhanced concentrations can possibly be related to geological rock formation (Kol, 1968). High concentrations of dissolved NH^+^_4_ in the snow fields of Spitsbergen could be related to the presence of moss vegetation (Table 1), because high NH^+^_4_ concentrations were found at sites 4/10 and 5/10 that received melt water from surrounding rock and moss substrata. A striking patchiness characterized the snow field communities on Spitsbergen (Figures 2A–E), with pronounced red tinted snow surrounded by bare uninhabited white snow. Although, within one snow field, we did not observe large differences in dissolved nutrient concentrations between totally differently colored snow communities, their cellular nutrient ratios revealed pronounced differences.

### Are snow algae N-limited?

Nitrogen is often considered a limiting nutrient for snow algae (Czygan, 1970) and other arctic microorganisms in cryoconite holes (Telling et al., 2011) as concentrations are often low and local enrichments might be mainly supplied from the faeces of birds (Müller et al., 2001; Fujii et al., 2010). In a study on snow algae in the Antarctic site, Langhovde, the primary nitrogen source appeared to be supplied from birds, and the authors concluded that an avian presence may affect red snow distribution significantly (Fujii et al., 2010). This seems to be consistent with the distribution of red snow in Antarctica, which is restricted to shore areas (e.g., Ling, 1996). In a snow algal study in the Antarctic the presence of green snow was predominantly detected in concert with high NH^+^_4_ concentrations, whereas the locations with red snow contained lower concentrations (Bidigare et al., 1993). In cryoconite holes, nitrogen might be the growth limiting nutrient, as rather low N concentrations (Säwström et al., 2007) and enhanced nitrogen fixation rates were determined on arctic glaciers in Svalbard (Telling et al., 2011). Our study contrasts with these studies, as the highest NH^+^_4_ concentrations were observed at sites inhabited by algae that largely differed in their C:N:P ratio, with no clear indication for a N-limitation (Figure 5). In addition, the presence of red snow did not coincide with the lowest dissolved N-concentrations (Figure 4). As a consequence, no indication for a (single) N-limitation based on dissolved and cellular N concentrations was found in our Arctic snow field communities.

In snow algae, a N-limitation greatly favors the accumulation of carotenoids (Britton et al., 1998) and lipids, which we also showed via the enhanced total FA content in three of the four snow algal strains in response to −N+HL (Table 2). In addition, the FA composition of the −N+HL strains more closely compared to the field samples than the +N+ML strain (Figure 6). Although, N-limitation combined with HL conditions changed the cultures from green into red or orange resting forms with thicker cell walls, this did not form the same cysts as found in the field, and the exact triggering mechanism that completes this process in the life cycle of snow algae is not yet fully understood. In other freshwater algae, enhanced lipid and FA accumulation have been reported in response to a N-limitation and HL conditions (e.g., Rodolfi et al., 2009; Cho et al., 2011; Piepho et al., 2012) and in some cases this is in conjunction with the accumulation of carotenoids. To induce cyst formation in the culture strains, we applied a nutrient-limitation in concert with HL conditions, which resulted in excess production and storage of lipids in several species of microalgae (Solovchenko et al., 2008; Piepho et al., 2012). It is therefore astonishing that at the site with the highest concentrations of nitrogen (5/10-1a) we found only some motile green flagellates, but the dominant forms were red cysts (>95%). The snow algal community at this N-rich site appeared just as red as at sites where snow contained no detectable N-concentrations! Obviously, N was not the major or only nutrient/factor in play.

### Are snow algae P-limited?

In some studies, P is considered the limiting nutrient for microbial communities on glacier surfaces (Säwström et al., 2007; Stibal et al., 2009), although in a study comparing 50 different fields with snow algal blooms, P concentrations in the algal cells was relatively high, suggesting sufficient availability (Müller et al., 1998). In our study, we only found a C:P ratio at one site (5/10-1a) high enough to suggest a possible P-limitation (i.e., 3-fold above the C:P ratio of 258 indicated for freshwater phytoplankton, North et al., 2007). At most sites, the C:P ratio did not exceed a molar ratio of 350, and taking into account the higher cellular C content in snow algae during the process of cyst formation, these values do not suggest P to be a major limiting nutrient. Interestingly, only at site 5/10 with the higher C:P ratio, were detectable concentrations of SRP found, and these were still low compared to values found in mountain snow (Hoham and Mullet, 1977). A possible argument that P might have been limiting at site 5/10-1a, is the high concentration of NH^+^_4_ that supplied the algae with sufficient N, but not enough P. Although less frequently reported, a P-limitation also often results in an enhanced FA content (Spijkerman and Wacker, 2011; Piepho et al., 2012), which might explain the similar FA accumulation and the red color of the snow at this site. As concluded for N, PO^3−^_4_ did not appear to be the (sole) nutrient limiting our Arctic snow algal communities.

The concentrations of NH^+^_4_ and SRP were highest at site 5/10 (Figure 4), possibly due to the larger numbers of overflying birds [and thus faeces, see Müller et al. (2001)]. Our data thus suggest that bird colonies can be an important source for nutrients, especially for phosphorus. However, the presence of birds is not the sole decisive factor for the occurrence of snow algal blooms.

### Other factors

The N:P ratio of the field samples resembles the Redfield ratio suggesting that N and P content are in balance. Therefore cellular ratios suggest that neither N or P limited the photosynthesis of the snow algae as a single nutrient. This does not exclude co-limitation of the algae by the two nutrients, possibly in concert with other factors, e.g., CO_2_, light, temperature, and topographic parameters such as the slope of the snowfield. In snow, a high C-demand might well result in inorganic C-limited photosynthesis as inorganic carbon concentrations will be low in the slightly acidic snow (pH ranging between 4.4 and 6.2; Müller et al., 1998). The pH values in snow are typically slightly acidic as a result of a limited buffering capacity and this low pH results in an absence or low concentration of bicarbonate (Stumm and Morgan, 1996). Thus, CO_2_ will be the most important inorganic carbon source, and its concentration might be limiting photosynthesis and growth of snow algae, especially in sub-surface snow where diffusion processes are decreased. Apart from the different *Chlamydomonas* spp., many green microalgae that live in snow belong to the genus *Chloromonas* and do not have a pyrenoid, supposedly resulting in a less efficient CO_2_ concentrating mechanism (Morita et al., 1999). For several phytoplankton species it has been shown that co-limiting or multiple stress factors might interact on the cellular C:N:P ratio and FA composition (Spijkerman and Wacker, 2011; Piepho et al., 2012). Alternatively, instead of studying the FA content and composition, nutrient-limiting conditions could have been more pronounced in changes in the lipid headgroups (van Mooy et al., 2009) or in changes in sterol composition (Piepho et al., 2010).

The deviation from the Redfield ratio might be caused by a high cellular C content, enhanced as a result of the high cellular starch, lipid and FA content, but might be decreased by low CO_2_ concentrations. Compared to four species of freshwater phytoplankton and *Chlamydomonas reinhardtii*, total FA content expressed on carbon was, however, not enhanced in the snow algal communities (values in general ranging between 100 and 400 mg FAtot g C^−1^; Piepho et al., 2012; Wacker and Martin-Creuzburg, 2012). In contrast, our values from the snow communities were relatively high when compared to *Chlorella* sp. that only contained 12.5–75 mg FAtot g C^−1^ (assuming that dry weight accounts for 50% of C; Cho et al., 2011), and also when compared with the marine microalga *Nanochloropsis* sp. that contained 75–165 mg FAtot g C^−1^ in outdoor cultures (Rodolfi et al., 2009). FA concentrations in the snow field communities were therefore not enhanced but rather covered the broad range found in other studies.

### The importance of slope

Nutrient mobilization due to snow melt is possibly important for the microorganism community on snow and glaciers. On a Greenland glacier, slope was considered the most ecologically significant variable, directly connected with snowmelt (Stibal et al., 2012). Stibal and colleagues even concluded that the effect of chemistry on net ecosystem production may be neglected and that physical factors dominate these processes. Indeed, slope might also have been an important parameter in our study, as concentrations of nutrients in the snow were highest in the snow field with the lowest angle (Table 1). Slope is a proxy for melting water rivulets, and in this context we can add observations from an inland glacier surface (Doktorbreen: Nathorst Land, N 77.566667°, E 016.900000°) of Spitsbergen which exhibited an intense red snow algal bloom with cell densities of 8.6 × 10^5^ cells ml^−1^ covering several tens of square meters (determined during an expedition in KOL/2004, unpublished results). The snow field was located far away from the coastline (approximately 25 km from the eastern coast of Spitsbergen) and had no bird colonies close by. The only input of inorganic minerals would have come from ice- and snow-free hill tops in the vicinity. Striking at this site, was the fact that the snow field was almost level and the snow was rather wet, thus supporting the importance of slope and melting water rivulets. We therefore support the suggestion that slope can be an important geographical and physical parameter that triggers the growth of snow algal communities restricted to localities with a favorable microhabitat.

### Fatty acid composition

The general pattern in FA composition of all snow communities studied, showed that the MUFA C18:1n-9 was the dominant FA. In the study of Bidigare et al. (1993) especially large differences were detected in the percentage of C16:0, C18:0, and C18:1n-9, with a nearly 60% FA content of the latter in red snow. Independent from the color of the snow algal community, this MUFA was the dominant FA in all our samples. In our laboratory strains, the concentration of oleic acid (C18:1n-9) was higher in −N+HL than in +N+ML conditions. Additionally, in other snow fields, carotenoid-rich red resting stages contained high concentrations of C18:1n-9 (Tazaki et al., 1994; Řezanka et al., 2008). The same FA increased in concentration in several species upon nutrient limitation and HL adaptation (Solovchenko et al., 2008; Rodolfi et al., 2009; Piepho et al., 2012 and references therein) and also in response to an acclimation to lower temperatures (Hochachka and Somero, 2002). Also, in *Chlamydomonas acidophila*, C18:1n-9 steadily increased with increasing P and C co-limiting conditions (Spijkerman and Wacker, 2011). Although this MUFA is an important precursor for many PUFAs, its accumulation under growth limiting conditions might not be a prerequisite to support cell physiology, but be an accumulation effect resulting from ceased growth. In culture experiments, a nutrient limitation cannot be uncoupled from decreased growth rates, and on our snow fields the algae were also preparing for overwintering by stopping cell proliferation and forming cysts. The extent of accumulation might differ between species due to differences in their life cycle, e.g., when preparing for overwintering.

The FA composition of the four +N+ML and −N+HL snow algal strains changed in response to the culture conditions in most strains. However, there were also large differences that partly resulted from species belonging to different taxonomic classes, namely the Chlorophyceae and Trebouxiophyceae and also within the genus *Chloromonas* (strains CCCryo 005-99 and CCCryo 010-99). Upon a N-limitation and at HL, both strains enhanced their total FA content nearly 2-fold, but in strain CCCryo 005-99 (*Cr. nivalis*) this was mainly a (6-fold) increase in the concentration of the PUFA C18:3n-3, whereas in strain CCCryo 010-99 (*Cr*. cf. *rostafinskii*) it was mainly a (5-fold) increase in the MUFA C18:1n-9. Species composition and differences in adaptation to nutrients and light might therefore largely affect the FA composition in a snow algal bloom.

### Total fatty acid content and unsaturation content

In laboratory cultures of snow algal strains as well as in field communities we found a similar range of total FA concentrations and high percentages of unsaturated FAs (up to 85% of total FA content). In different species of algae, the total FA content often increased when growth ceased due to C-, N-, and/or P- limitation, but also it increased with increasing growth light intensity (Piepho et al., 2012). For example, in cultures of *Nannochloropsis* sp. total FA content related to biomass increased from 13 to 50% upon the onset of a N- or P-limitation (Rodolfi et al., 2009). In batch cultures of *Chlorella* sp., any limiting condition and thus ceasing growth rate, resulted in enhanced FA content over time (Cho et al., 2011). In *Chlamydomonas acidophila*, the total FA content increased 3-fold when the cellular P quota decreased 4-fold (Spijkerman and Wacker, 2011). Nevertheless, as not all of the four snow algal strains tested in this study increased their total FA content under −N+HL conditions (Table 2) adjustment of total FA appears to be a species-specific reaction.

In Antarctic red snow from Hermit Island (Antarctica) the total FA content was similar to snow predominantly inhabited by green forms (Bidigare et al., 1993). However, the composition of the FAs was largely different as FAs in green cells consisted mainly of saturated FAs (72%) whereas in red cells unsaturated FAs (with 80%) clearly presided over saturated FAs (Bidigare et al., 1993). We also found high percentages of unsaturated FAs in the snow algal communities which composition of orange, red or green cells did not result in different FA profiles. The FA unsaturation found in the snow algal communities were comparable with a high proportion of PUFAs (75%) in the snow alga *Chloromonas brevispina* (Řezanka et al., 2008). In contrast, from our two strains of *Chloromonas*, only strain CCCryo 010-99 was rich in PUFAs (Table 2), whereas strain CCCryo 005-99 was only rich in PUFAs under −N+HL. In snow algae communities of the Antarctic, low concentrations of the PUFA C18:3n-3 have been reported (Bidigare et al., 1993), a PUFA that was highly abundant in our snow communities (Figure 6A). While the low concentrations in the Antarctic samples might result from a relative high abundance of diatoms that have a low C18:3n-3 content (Teoh et al., 2004; Piepho et al., 2012), the high C18:3n-3 concentrations in our arctic samples originated from the dominance of C18:3n-3-rich green algal species. The presence of other algal species, consumers, or consumer resting stages will contribute to the recovery of long-chained FAs (C22; Desvilettes and Bec, 2009) that were not found in our four strains, but were present in the field samples. In general, temperature is a very important parameter, determining the unsaturation percentage, as an increase of PUFAs in algae grown under conditions with decreasing temperature has been shown in several species (Poerschmann et al., 2004; Teoh et al., 2004; Piepho et al., 2012). A general pattern in PUFA content related to growth temperature was not found in six Antarctic microalgae (Teoh et al., 2004), and also in our study, temperature differences were considered less important, as we found large differences in PUFA content between the four arctic strains cultured under +N+ML and −N+HL conditions at the same temperature. Individual species dominance on snow fields will likely have an important effect on FA content and composition, independent of differences in nutrient limitation, light and temperature.

In conclusion, no single nutrient limitation, or physical or geological factor can fully explain the occurrence and color of snow algal blooms and only a suite of physical and geological factors in concert with ecophysiological adaptations might explain the persistent establishment of this impressive phenomenon of tinted snow.

### Conflict of interest statement

Part of this research was supported by the German Science Foundation which had no role in study design, data collection and analysis, decision to publish, or preparation of the manuscript.
